# RAD54 N-terminal domain is a DNA sensor that couples ATP hydrolysis with branch migration of Holliday junctions

**DOI:** 10.1038/s41467-017-02497-x

**Published:** 2018-01-02

**Authors:** Nadish Goyal, Matthew J. Rossi, Olga M. Mazina, Yong Chi, Robert L. Moritz, Bruce E. Clurman, Alexander V. Mazin

**Affiliations:** 1 Department of Biochemistry and Molecular Biology, Drexel University College of Medicine, Philadelphia, PA 19102 USA; 2 Divisions of Clinical Research and Human Biology, Fred Hutchinson Cancer Research Center, Seattle, WA 98109 USA; 3 Institute for Systems Biology, Seattle, WA 98109 USA

## Abstract

In eukaryotes, RAD54 catalyzes branch migration (BM) of Holliday junctions, a basic process during DNA repair, replication, and recombination. RAD54 also stimulates RAD51 recombinase and has other activities. Here, we investigate the structural determinants for different RAD54 activities. We find that the RAD54 N-terminal domain (NTD) is responsible for initiation of BM through two coupled, but distinct steps; specific binding to Holliday junctions and RAD54 oligomerization. Furthermore, we find that the RAD54 oligomeric state can be controlled by NTD phosphorylation at S49, a CDK2 consensus site, which inhibits RAD54 oligomerization and, consequently, BM. Importantly, the effect of phosphorylation on RAD54 oligomerization is specific for BM, as it does not affect stimulation of RAD51 recombinase by RAD54. Thus, the transition of the oligomeric states provides an important control of the biological functions of RAD54 and, likely, other multifunctional proteins.

## Abstract (web summary)

RAD54 stimulates activity of the RAD51 recombinase and catalyzes branch migration of Holliday junctions during DNA repair and recombination. Here the authors show that the N-terminal domain of RAD54 mediates RAD54 oligomerization to promote branch migration, and is the target of phosphorylation that inhibits oligomerization and branch migration but not RAD51 stimulation.

## Introduction

The HR pathway is responsible for the repair of DNA double-strand breaks (DSBs), the most harmful types of DNA lesions, faithful chromosome segregation during meiosis, and telomerase-independent telomere maintenance^1–3^. HR uses homologous DNA molecules as a template to repair DSBs and therefore is, generally, an error-free process. During DSB repair by HR, the dsDNA ends undergo exonucleolytic resection to generate protruding ssDNA tails^4^. RAD51 recombinase binds to the ssDNA tails forming a nucleoprotein filament that performs a search for homologous dsDNA^5^. The RAD51-ssDNA filament then invades the homologous dsDNA generating joint molecules (D-loops) that further extend into the DNA four-way cross-structure known as a Holliday Junction (HJ)^6–8^. The HJ has a remarkable ability to translocate along the DNA axis through a process known as branch migration (BM), in which one strand of the DNA duplex became progressively exchanged for the homologous strand of another DNA duplex by the stepwise breakage and reformation of base pairs. In different HR mechanisms, BM may cause either dissociation or extension of joint molecules. It may also promote a restart of DNA replication stalled at a DNA damage site by switching DNA-template strands through a reversible regression of replication forks into Holliday junctions^9^.

Previously, we showed that RAD54, a member of the Rad52 epistasis group^10,11^, promotes BM of HJ^9,12,13^. BM activity of RAD54 requires ATP hydrolysis and involves the formation of higher order RAD54 oligomers on HJ-like structures^12,14^. RAD54 promotes BM with significantly greater efficiency than other known eukaryotic BM proteins, like BLM or RECQ1^13^. Moreover, similar to RuvAB, a prototypical BM protein from *E. coli*, RAD54 is capable of driving BM of HJ, through large regions of DNA sequence heterology^13^.

**Fig. 1:**
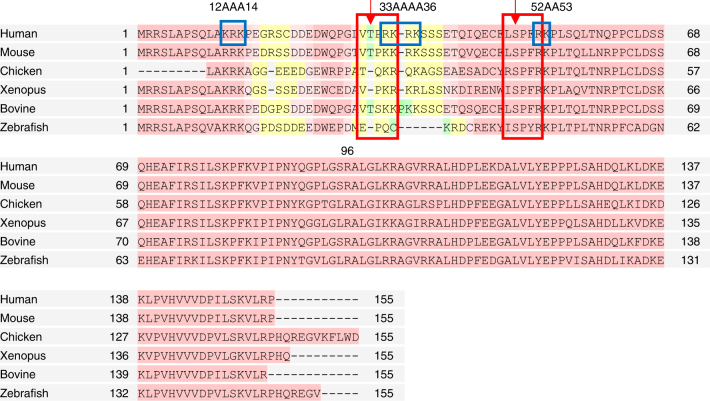
The Rad54 NTD a.a. sequences from human (*Homo sapiens*), mouse (*Mus musculus*), chicken (*Gallus gallus*), xenopus (*Xenopus tropicalis*), bovine (*Bos* *taurus*) and zebrafish (*Danio rerio*) were analyzed using multiple sequence alignment program, T-coffee^49^. Pink, yellow, and green colored regions show high, low, and very low conservation among sequences, respectively. The red box highlights the CDK2 phosphorylation consensus sequence with arrows pointing to the residue that became phosphorylated. The blue box highlights the basic residues that were mutated to alanines with the name of the mutants indicated above the box. Residue 96 marks the first a.a. residue of the RAD54_96__–__747_ truncated mutant

RAD54 belongs to the SWI/SNF2 family of helicase-like proteins of Superfamily (SF) 2 helicases^15,16^. Similar to other known SWI/SNF proteins, but unlike canonical helicases, RAD54 does not have DNA strand-separation activity. Structurally, RAD54 is composed of the N-terminal domain (NTD) (Fig. 1), the ATPase/DNA-binding core domain, and the C-terminal domain (CTD). The ATPase/DNA-binding core domain contains seven motifs conserved in all SF2 and SF1 proteins, which constitute the two tandem RecA-like folds responsible for protein binding to DNA and translocation in an ATPase-dependent manner^17–19^. The NTD (Fig. 1) and CTD are conserved among RAD54 orthologs and their functional role remains to be elucidated. The RAD54 NTD contains two CDK2 consensus motifs indicating a regulatory role of this domain (Fig. 1).

RAD54 is a multifunctional protein that, in addition to BM, can physically interact with RAD51^20,21^ and stimulate its DNA strand exchange activity^22–24^, it can also displace nucleosomes^25–27^ or RAD51–dsDNA complexes^28^. However, the structural basis for different activities of RAD54 is currently unknown.

In this study, using the N-terminal truncated RAD54_96–747_, we found that the NTD of RAD54 is essential for its BM activity. At the same time, the RAD54_96–747_ retains the ability to translocate on dsDNA in an ATPase-dependent manner, indicating a specific role of the RAD54 NTD in BM. We found that a distinct DNA-binding site located in the NTD (Fig. 1)^29^ is critical for RAD54 BM. First, this site binds HJ and HJ-like structures with high affinity. Second, DNA binding by this site enables protein oligomerization that is required for BM. We identified a specific amino-acid residue, S49, which is important for protein oligomerization. Furthermore, we show that CDK2 phosphorylation at S49 in vitro or the S49E phosphomimetic mutation impairs DNA-dependent RAD54 oligomerization and inhibits RAD54 BM activity. Importantly, CDK2 phosphorylation has no inhibitory effect on stimulation of RAD51 recombinase by RAD54. Taking together, these data indicate that the RAD54 oligomerization is a controlled step that has a pivotal role in the regulation of the protein activities.

## Results

### The RAD54 NTD is essential for BM of Holliday junctions

**Fig. 2:**
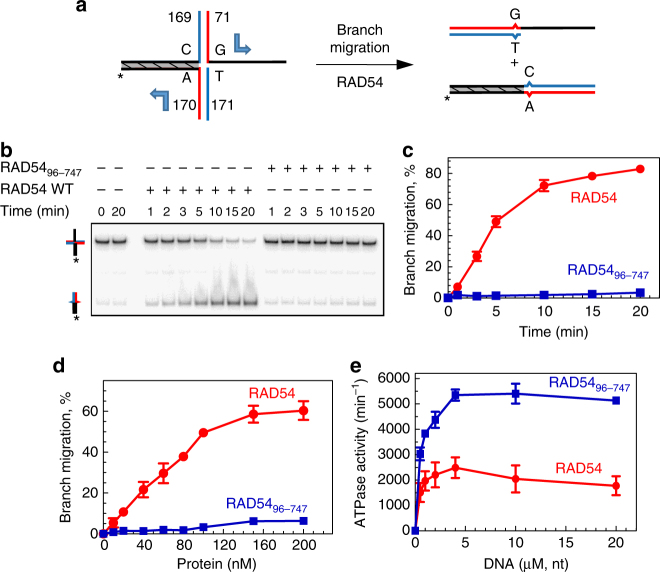
The RAD54 NTD is essential for BM, but not for ATP hydrolysis. **a** The experimental scheme of the BM reaction. Shown is one bp heterology that was introduced into the PX junction (no. 71/169/170/171) to reduce the spontaneous BM. The shaded region denotes the heterologous DNA branch. The direction of BM is denoted by blue arrows. The asterisk indicates the ^32^P-label. The numbers correspond to oligonucleotides in Supplementary Table 1. **b** The kinetics of BM of PX junction (10 nM, molecules) promoted by RAD54 (30 nM) or RAD54_96–747_ (30 nM). The BM products were analyzed by electrophoresis in 8% polyacrylamide gels. **c** Data from **b** are plotted as a graph. **d** The effect of RAD54 or RAD54_96–747_ concentrations on DNA BM. The reaction was carried out for 90 s. **e** ATP hydrolysis by RAD54 (20 nM) and RAD54_96–747_ (20 nM) using supercoiled pUC19 as DNA substrate. Each experiment was repeated three times. Error bars represent the standard error of the mean (s.e.m.)

We wished to explore the role of the NTD in the BM activity of RAD54. We constructed a truncated RAD54_96–747_ mutant devoid of the N-terminal 95 amino-acid (a.a.) residues and tested its BM activity using ^32^P-labeled partial Holliday junctions (PX) substrate (no. 71/169/170/171) that contains three dsDNA arms and one ssDNA arm (Fig. 2a). Previously, we showed that the PX junction is a preferable substrate for RAD54 BM^12,14^. To reduce spontaneous BM, a single bp of heterology was introduced in one of the four DNA arms, which during BM would create a mismatched bp in each of the two BM products (Fig. 2a). We found that the RAD54_96–747_ mutant has no detectable BM activity in a range of tested protein concentrations (Fig. 2b–d; Supplementary Figure 1a). As BM activity of RAD54 depends on ATP hydrolysis^12^, we tested whether the loss of BM activity by RAD54_96–747_ is due to disruption of its ATPase activity. Surprisingly, we found that in the presence of supercoiled pUC19 DNA the RAD54_96–747_ has an ~2–2.5-fold greater ATPase activity than RAD54 WT (Fig. 2e). Thus, the NTD truncation specifically disrupts the BM, but not the ATPase activity of RAD54.

### RAD54_96–747_ retains DNA translocation activity

**Fig. 3:**
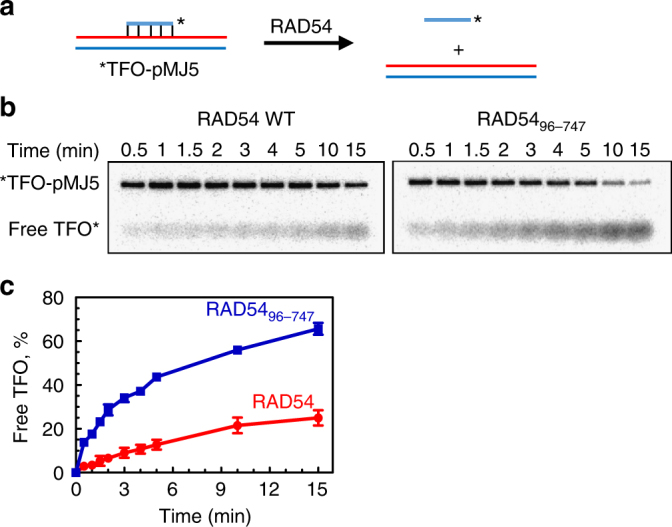
The 95 a.a. N-terminal truncation enhances DNA translocation activity of RAD54. **a** The experimental scheme of the triple-helix displacement assay. The triple-helix forming oligonucleotide (TFO) is paired to the linearized pMJ5 plasmid. The asterisk indicates the ^32^P-label. **b** The kinetics of triple-helix (0.5 nM, molecules) displacement by RAD54 (5 nM) or RAD54_96–747_ (5 nM) was analyzed by electrophoresis in 1.2 % agarose gels. **c** Data from **b** are plotted as a graph. The values obtained in protein-free reaction (Supplementary Figure 1b) were subtracted from the values of RAD54-promoted reactions. Each experiment was repeated three times. Error bars represent the s.e.m.

At minimum, a BM protein must translocate along the DNA axis in an ATPase-dependent manner. We wanted to test whether RAD54_96–747_ retains DNA translocation activity using a triple-helix displacement assay (Fig. 3a)^30–32^. The triple-helix was formed under slightly acidic conditions, pH 5.5, by annealing a pyrimidine-rich triplex-forming oligonucleotide (TFO, 22 mer) to a purine-rich sequence in pMJ5 plasmid DNA through Hoogsteen base pairing. The triple-helix is stable at neutral pH, however once disrupted by a DNA translocating protein, it will not re-form. We found that the RAD54_96–747_ retained DNA translocation activity, moreover, it was even increased, ~2–2.5-fold, relative to that of RAD54 WT (Fig. 3b, c; Supplementary Figure 1b). The increase of DNA translocation activity of RAD54_96–747_ was in parallel with the increase in its ATPase activity but in a sharp contrast with the loss of the BM activity (Fig. 2).

### The RAD54 NTD binds preferentially to branched DNA

**Fig. 4:**
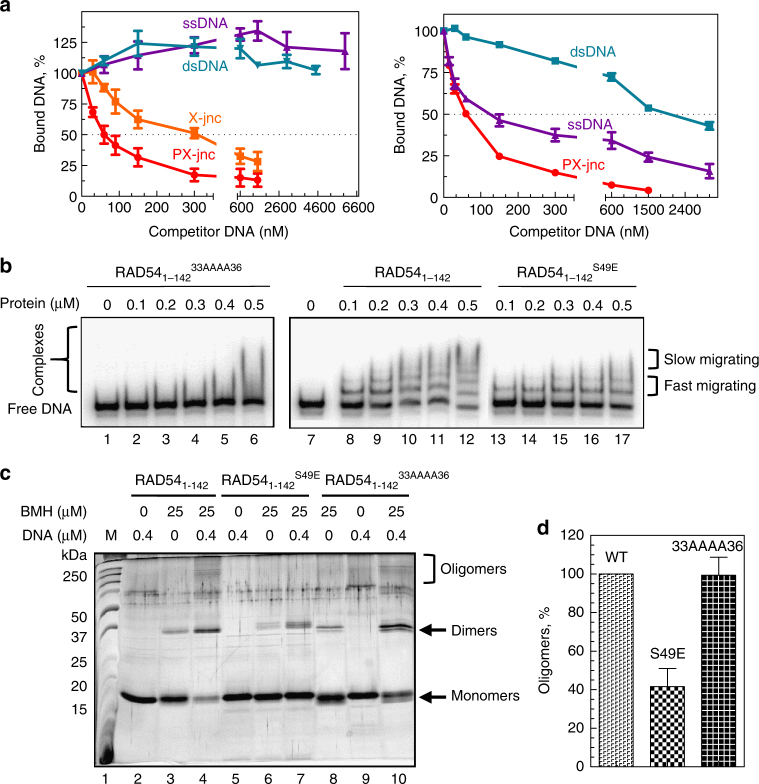
The DNA-binding properties of RAD54_1–142_. **a** RAD54_1–142_ (300 nM) or RAD54_156–747_ (100 nM) was incubated with ^32^P-labeled non-mobile PX junction (no. 174/175/176/181; 30 nM) in the presence of the indicated concentrations of unlabeled DNA competitors. The complexes were analyzed by EMSA. **b** The effect of the S49E and 33AAAA36 mutations on RAD54_1–142_ binding to PX junction (no. 174/175/176/181; 30 nM) was analyzed by EMSA in a 6% polyacrylamide gel. **c** The S49E, but not the 33AAAA36, mutation inhibits DNA-dependent oligomerization of RAD54_1–142._ The proteins (1.2 µM) were incubated with or without the flap DNA (no. 244/249/250; 0.4 µM) in the presence or absence of BMH (25 µM) and analyzed in a 15% SDS-PAGE. Arrows indicate migration of the monomeric, dimeric, and oligomeric protein products. The molecular weight standards (Precision Plus; Bio-Rad) are shown. **d** The relative fractions of the oligomers in **c** were quantified and presented as a graph. Each experiment was repeated three times. Error bars represent the s.e.m.

In addition to the DNA-binding site in the ATPase core domain, RAD54 has a secondary DNA-binding site in the NTD^29^. To assess a possible role of this site in RAD54 BM of Holliday junctions, we purified two constructs, the RAD54_1–142_ NTD^33^ and RAD54_156–747_ lacking the NTD from *E. coli* and studied their DNA-binding properties. We analyzed formation RAD54_1–142_ complexes with ^32^P-labeled PX junction (no. 174/175/176/181) in the presence of increasing concentrations of unlabeled DNA competitors of different structures using EMSA. Similar to RAD54 WT^12^, we found that RAD54_1–142_ shows a strong binding preference for branched DNA. RAD54_1–142_ complexes with PX junctions (no. 174/175/176/181) were stable in the presence of a 150–200-fold excess of cold ss- or dsDNA (no. 2 or no. 1/2) competitor (Fig. 4a, left panel; Supplementary Figure 2a). Also, similar to RAD54 WT, RAD54_1–142_ has a strong, approximately six-fold, preference for PX junction over HJ-junction substrates. In contrast, RAD54_156–747_ lost the preference for HJ-like structures; in the presence of a four-fold excess of ssDNA, 50% of the RAD54_156–747_ complexes with PX junctions dissociated (Fig. 4a, right panel; Supplementary Figure 2b). These data suggest that the NTD DNA-binding site may have an important role in determining the preferential binding of RAD54 to HJ-like structures.

### Mutations that impair NTD oligomerization or DNA binding

To identify the specific a.a. residues essential for DNA binding of RAD54_1–142_, we generated three mutants: RAD54_1–142_^12AAA14^ (KRK residues at position 12–14 mutated to alanines), RAD54_1–142_^33AAAA36^ (RKRK residues at position 33–36 mutated to alanines), and RAD54_1–142_^52AA53^ (RK residues at position 52–53 mutated to alanines) (Fig. 1). We also constructed RAD54_1–142_^T31E^ and RAD54_1–142_^S49E^ phosphomimetic mutants, as the RAD54 NTD was found to be phosphorylated in mitotic HeLa cells on T31 and S49^34^, each within a CDK consensus motif X(S/T*)PX(K/R) (Fig. 1). We wanted to test the effect of phosphorylation at these sites on DNA binding by the RAD54 NTD. All RAD54 NTD mutant proteins, except RAD54_1–142_^12AAA14^ that failed to express under various tested growth conditions, were purified from *E. coli*, and examined for binding to PX junction (no. 174/175/176/181) using EMSA.

RAD54_1–142_^52AA53^ bound DNA similar to the non-mutated RAD54_1–142_ that formed a series of distinct complexes starting at 0.1 µM protein concentration (Fig. 4b, lanes 8–12). In contrast, RAD54_1–142_^33AAAA36^ showed only weak binding to PX junctions. No distinct complexes were observed on the gel, however, smearing observed at the highest protein concentration tested (0.5 µM) likely indicated the formation of unstable DNA–protein complexes (Fig. 4b, lanes 1–6; Supplementary Figure 2d).

The S49E mutation reduced DNA binding of RAD54_1–142_; specifically, it reduced the formation of large nucleoprotein complexes, whereas formation of small fast migrating complexes was not significantly affected (Fig. 4b, lanes 13–17; Supplementary Figure 2d). In contrast, the T31E mutation had no significant effect on DNA binding by RAD54_1–142_ (Supplementary Figure 2c, d). Thus, both the 33AAAA36 and S49E mutations reduced DNA binding of RAD54_1–142_, and the latter mutation specifically disrupted formation of large nucleoprotein complexes.

In solution, RAD54 is a monomer, but it oligomerizes in the presence of DNA^35^. Previously, we suggested that DNA-dependent RAD54 oligomerization is necessary for BM^14^. To test if either of the two NTD mutations, 33AAAA36 or S49E, affect the RAD54 oligomerization, we performed cross-linking experiments using BMH agent that links two cysteine residues in the range of distances 3.5–15.6 Å^36^. RAD54_1–142_, RAD54_1__–__142_^S49E^ or RAD54_1–142_^33AAAA36^ were incubated with the “minimal” DNA flap substrate (no. 244/249/250) containing two 15-bp dsDNA arms and one 45-nt ssDNA arm, the smallest DNA substrate that is capable to efficiently stimulate the ATPase activity of RAD54^14^ followed by BMH cross-linking. The products of the protein cross-linking were analyzed on an SDS-polyacrylamide gel. Untreated RAD54_1–142_, RAD54_1–142_^S49E^, and RAD54_1–142_^33AAAA36^ migrated in accord with the predicted size (~16 kDa) (Fig. 4c, lanes 2, 5, and 9). In the absence of DNA, BMH treatment gave rise to a small additional band of ~36 kDa, consistent with predicted dimer size (32 kDa) (Fig. 4c, lanes 3, 6, and 8). In the presence of DNA, BMH cross-linking resulted in a larger fraction of dimers (Fig. 4c, lanes 4, 7, and 10). For RAD54_1–142_ and RAD54_1–142_^33AAAA36^, large oligomers were also observed, some of them were unable to enter the separating gel (Fig. 4c, lanes 4 and 10). Apparently, BMH cross-linking helped to visualize unstable nucleoprotein complexes formed by RAD54_1–142_^33AAAA36^ (Fig. 4b; lanes 1–6). In contrast, higher order oligomers were nearly completely absent in RAD54_1–142_^S49E^ (Fig. 4c, lane 7).

Thus, S49E and 33AAAA36 mutations differently affect the interaction of the RAD54 NTD with PX junctions. The S49E did not significantly disrupt the formation of fast migrating complexes in EMSA and protein dimers in cross-linking experiments, but it markedly decreased the formation of large protein oligomers (Fig. 4d). In contrast, 33AAAA36 did not affect DNA-dependent oligomerization of the RAD54 NTD, but it significantly reduced the formation of stable protein–DNA complexes visualized by EMSA (Fig. 4b).

### The NTD mutations inhibit RAD54 BM

**Fig. 5:**
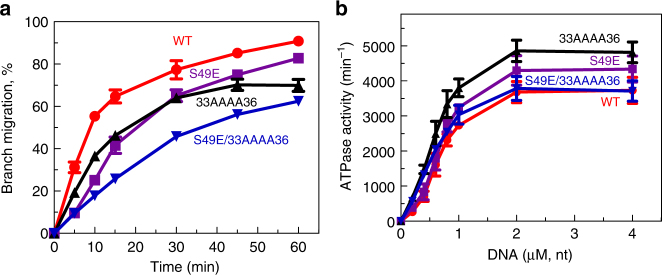
The S49E and 33AAAA36 mutations inhibit BM, but not ATP hydrolysis. **a** The effect of the S49E and 33AAAA36 single and double mutations on the kinetics of BM of PX junction (no. 71/169/170/171; 32 nM) by RAD54 (60 nM). **b** The effect of the S49E and 33AAAA36 mutations on ATP hydrolysis by RAD54 (20 nM) using supercoiled pUC19 as DNA substrate. Each experiment was repeated three times. Error bars represent the s.e.m.

Next, we endeavored to test the effect of the 33AAAA36 and S49E mutations on the BM activity of RAD54. We constructed full-length RAD54 carrying these NTD mutations with a sumo-His tag and purified them from *E. coli*. In control experiments, the ATPase and BM activities of RAD54 WT with this tag purified from *E. coli* were similar to that of RAD54 purified from insect cells (Supplementary Figure 3a, b). Using PX junctions (no. 71/169/170/171) as a substrate, we found that the initial rate of BM for RAD54^33AAAA36^ and RAD54^S49E^ was reduced by ~1.7-fold and 2.5-fold, respectively. (Fig. 5a). This inhibition could not be due to a decrease in the ATPase activity that was slightly elevated in both mutants relative to RAD54 WT (Fig. 5b). Thus, the mutations that either disrupted DNA binding of the NTD or inhibited its oligomerization caused inhibition of RAD54 BM activity.

If two mutations inhibit BM by different mechanisms, one could expect that their combination would inhibit BM stronger than any of them individually. Indeed, we found that in the double mutant that the inhibition of BM activity was stronger than in either of the single mutants (Fig. 5a) consistent with the different mechanisms of inhibition caused by these mutations: DNA binding and protein oligomerization.

### RAD54 NTD phosphorylation by CDK2 specifically inhibits BM

Because RAD54 oligomerization, which is required for BM, is reduced in the S49E phosphomimetic mutant, we next examined the effect of the NTD phosphorylation on the RAD54 BM activity. We first tested whether recombinant CDK2/cyclinE can phosphorylate RAD54 and the RAD54_1–142_ in vitro. We observed that both proteins were phosphorylated with similar efficiency, consistent with the position of two CDK2 consensuses in the RAD54 NTD (Supplementary Figure 4a). When we used the RAD54^S49E^ and RAD54^T31E,S49E^ mutants as substrates for CDK2/cyclinE, the level of phosphorylation was reduced to 65 and 15% for the single and double mutant, respectively, indicating that T31 and S49 are the major phosphorylation sites in RAD54 (Supplementary Figure 4b). These data were in agreement with the results of mass spectrometry analysis, which directly demonstrated that T31 and S49 were the two main sites of RAD54 phosphorylation by CDK2 in vitro with a small contribution of other phosphorylation sites (Supplementary Figure 5a).

**Fig. 6:**
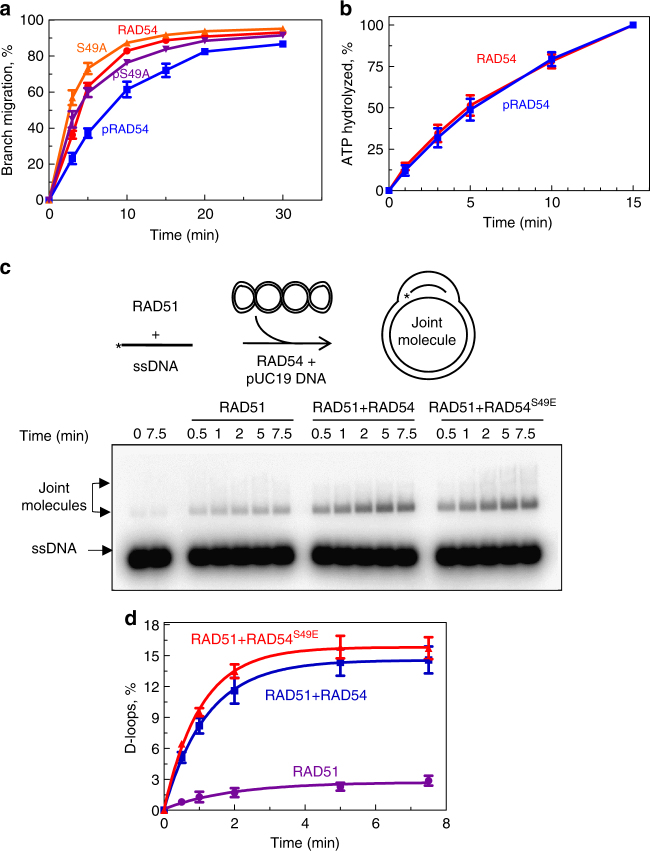
Effect of CDK2 phosphorylation on the RAD54 activities. **a** The kinetics of BM of PX junction (no. 71/169/170/171; 10 nM) promoted by RAD54 (60 nM), phosphorylated RAD54 (60 nM), RAD54^S49A^ (60 nM), or phosphorylated RAD54^S49A^ (60 nM). **b** The effect of RAD54 phosphorylation (60 nM) on its ATPase activity was tested using supercoiled pUC19 (2 µM, nt) as DNA substrate. **c** The experimental scheme of the D-loop reaction. The nucleoprotein filaments were formed between RAD51 and ssDNA for 30 min at 37 °C. The reactions were then moved to 30 °C. Then RAD54 was added followed by addition of pUC19 dsDNA substrate to initiate the reaction. The effect of RAD54 (50 nM) or phosphomimetic RAD54^S49E^ (50 nM) on D-loop formation by RAD51 (1.25 µM) between ^32^P-labeled ssDNA (no. 90; 2.4 µM nts) and pUC19 (50 µM, nts). The D-loops were analyzed by electrophoresis in 1% agarose gel. **d** Data from **c** are plotted as a graph. Each experiment was repeated three times. Error bars represent the s.e.m.

Next, we tested the effect of CDK2 phosphorylation on the BM activity of RAD54. We found that phosphorylation reduced RAD54 BM activity (Fig. 6a). In control with the RAD54^S49A^ mutant, we found that the S49A mutation has no significant effect on the RAD54 BM activity (Fig. 6a). Even when the RAD54^S49A^ mutant was subjected to phosphorylation by CDK2, the RAD54 BM was not affected (Fig. 6a). These results suggest that the effect of CDK2 on RAD54 BM was specific to phosphorylation of S49 residue. In contrast, we did not observe any effect of CDK2 phosphorylation on the ATPase activity of RAD54 (Fig. 6b). We also found that CDK2 phosphorylation did not inhibit RAD54 ability to stimulate DNA strand exchange activity of RAD51 (Fig. 6c, d). These data suggest that phosphorylation of S49 in the NTD may specifically regulate RAD54 BM activity.

## Discussion

RAD54 is an important HR protein that is evolutionarily conserved in eukaryotes. In vitro, it possesses several activities including stimulation of the RAD51 DNA strand exchange activity and remodeling dsDNA–protein complexes^7^. RAD54 stands out as the most proficient BM protein in eukaryotes known so far^7,12,13^. Moreover, RAD54 is the only known eukaryotic protein that is on par with *E.coli* RuvAB in its ability to bypass large regions of DNA heterology (~100 bp) during BM of HJ^13^. This property is remarkable because, in contrast to most other BM proteins, including RuvAB or BLM, RAD54 lacks canonical DNA helicase activity.

In spite of significant structural diversity, all BM proteins show high affinity to HJ or HJ-like structures and an ability to translocate on DNA in an ATPase-dependent manner. In the prototypic RuvAB complex, these two functions are segregated between RuvA and RuvB proteins, which act as the structure-specific DNA-binding protein and the ATPase motor protein, respectively^37,38^. However, RAD54 and other known BM proteins including bacterial RecG and eukaryotic RecQ family helicases, BLM, RECQ1, and WRN; combine these two important activities in a single polypeptide.

Our current data demonstrate that the NTD is essential for RAD54 BM activity. The RAD54_96–747_ mutant that lacks a significant portion of the NTD is almost completely devoid of BM activity, while still being proficient in DNA translocation and in ATP hydrolysis. We found that the RAD54 NTD promotes specific binding of RAD54 to HJ substrates. The RAD54_1–142_ NTD has a preference for HJ-like DNA substrates as strong as the full-length RAD54 and removing the NTD in the RAD54_156–747_ results in loss of preference for HJ-like substrates. Replacement of basic a.a. residues in the 33AAAA36 RAD54_1–142_ mutant by alanines diminished DNA binding by the RAD54 NTD indicating the tentative position of the NTD DNA-binding site, or at least a component of this site. Although the RAD54 NTD shows no structural homology outside of the RAD54 protein family, it may represent a functional analog of the NTD of RecG or the RecQ helicases CTD (RQC) that are responsible for specific binding to branched DNA structures^39–41^. Furthermore, deletion of the 95 a.a. N-terminal residues of the RAD54, similar to deletion of the BLM HRDC domain adjacent to RQC^42^, increased the ATPase activity of the enzyme. It is thought that HRDC may suppress the BLM ATPase activity through direct interaction with the ATPase core. Similarly, the RAD54 NTD may also interact with the ATPase core domain modulating its activity. However, this interaction may be transient, as we could not detect it in a pulldown assay.

The proteolytic cleavage analysis shows that the *S. cerevisiae* NTD Rad54 is unstructured in solution^21^. These data are consistent with the structural flexibility of the RAD54 NTD, which enables its interaction with various partners, like RAD51 or DNA^21,29,33^. Our current data further emphasize the structural flexibility of the RAD54 NTD by demonstrating a coupling between its binding to DNA and oligomerization. Previously, we showed that RAD54 forms oligomeric structures on HJ substrates during BM^14^, a property that is shared with other BM proteins including BLM and RuvAB^13,37^. Our current data show that RAD54 NTD is responsible for DNA-dependent RAD54 oligomerization. Moreover, we identified NTD mutations that separately affect DNA binding and protein oligomerization. Thus, 33AAAA36 mutation decreases DNA binding of the RAD54_1–142_ to DNA significantly. However, even this residual DNA binding appeared to be sufficient to induce protein oligomerization identified in BMH cross-linking experiments. In contrast, the S49E mutation only weakly inhibits initial binding of RAD54_1–142_ to DNA but disrupts the formation of large oligomeric protein complexes visualized by BMH cross-linking. In full-length RAD54, each of these mutations inhibited BM and their combination increased the inhibition.

Taken together, these data suggest a two-step process during formation of the active RAD54 BM complex. First, the RAD54 NTD specifically targets RAD54 to HJ. In the presence of Mg^2+^ (>100 µM), HJ exists in stacked conformation that is refractory to BM^14^. On the basis of structural data, it was recently proposed that binding of the BLM RQC domain induces structural transition of HJ into open conformation that is proficient in BM^40^. We suggest that binding of the RAD54 NTD may have a similar effect on the HJ conformation. Second, binding of the RAD54 NTD to DNA enables RAD54 oligomerization, which we show is required for BM.

A phosphoproteomics study identified RAD54 as a target for phosphorylation at S49^34^. Two CDK2 consensus sites are located in the RAD54 NTD, with putative phosphorylation sites at T31 and S49. In accord, our mass spec data identified T31 and S49 as major sites of RAD54 phosphorylation by CDK2 in vitro. Also, T31E, S49E double mutations significantly reduced CDK2 phosphorylation of RAD54. We found that S49E, but not the T31E phosphomimetic mutation, inhibits RAD54 NTD oligomerization. When incorporated into the full-length RAD54, this mutation inhibited BM activity; consistent with the important role of RAD54 oligomerization for BM. The effect of the S49E mutation is specific for BM, as it has no significant effect on stimulation of RAD51-promoted DNA strand exchange by RAD54, meaning RAD54 oligomerization is not required for stimulation of DNA strand exchange promoted by RAD51. In vitro, CDK2 phosphorylation of RAD54 also inhibited RAD54 BM showing an even stronger effect than the phosphomimetic S49E mutation. We suggest that CDK2 may regulate RAD54 BM activity in vivo. More work is needed to test the role of CDK2 in RAD54 regulation.

**Fig. 7:**
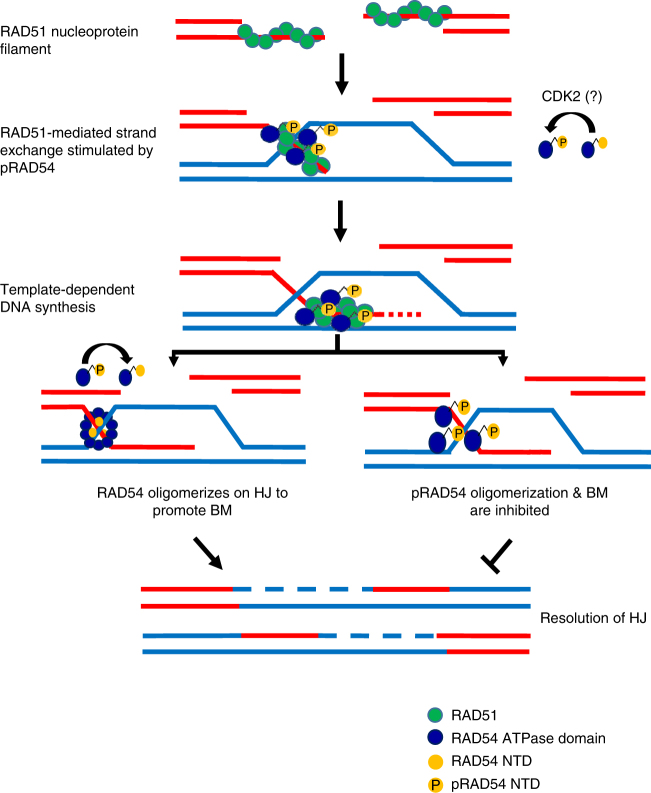
Proposed mechanism of regulation of the RAD54 activities. The initial steps of HR involve exonucleolytic processing of DSB ends producing 3′‐ssDNA overhangs, the formation of RAD51‐ssDNA nucleoprotein filaments, search for homology, and strand invasion into the homologous duplex DNA‐template leading to the formation of joint molecules (D‐loops). In its phosphorylated form, RAD54 can stimulate the strand exchange activity of RAD51. However, pRAD54 is unable to form an oligomer that is required for D-loop dissociation through BM; thus inhibiting RAD54 BM activity may provide sufficient time for the extension of the invading strand by a DNA polymerase. During later stages of HR, pRAD54 needs to be dephosphorylated so that BM could occur to complete the DSB repair process. Thus, phosphorylation modulates activities of RAD54 during HR

Previously, we showed that RAD54 BM activity causes dissociation of D-loops, the product of ssDNA invasion into homologous DNA-template during DSB repair^43^. This step of DSB repair, however, needs to be precisely controlled, as premature disruption of D-loops prior to their extension by DNA polymerase may abort DSB repair. CDK2-dependent inhibition of RAD54 BM may help to delay D-loop disruption until the polymerization step is completed (Fig. 7). Recently, it was shown that Nek1 phosphorylates RAD54 at S572 in late G2 phase to modulate its interaction with RAD51^44^. Thus, multiple mechanisms are in place to control different RAD54 activities in the cell.

In summary, we show that RAD54 NTD has a pivotal role in BM activity by specific binding to HJs and promoting DNA-dependent oligomerization. Furthermore, the RAD54 NTD, being a target for CDK2 phosphorylation, may have a role at the hub that controls RAD54 activities in the cell.

## Methods

### Proteins and DNA

GST-cyclinE-CDK2 was expressed in baculovirus-infected Sf9 cells and purified by ion-exchange and gel filtration chromatography^45^. All oligonucleotides used in this study (Supplementary Table 1) were purchased from IDT Inc and purified using denaturing 6–10% PAGE. To prepare oligonucleotide dsDNA substrates, complementary ssDNA oligonucleotides were annealed^46^ and stored at −20 °C. Oligonucleotides were labeled using [γ-^32^P] ATP and T4 polynucleotide kinase. T4 polynucleotide kinase and restriction endonucleases were purchased from New England Biolabs. PreScission™ Protease was purchased from GE Healthcare Life Sciences.

### Purification of RAD54 and RAD54 mutants from insect cells

Human RAD54 and RAD54_96–747_ were purified from Sf21 cells^24^. We used RAD54 constructs with an N-terminal GST tag and a PreScission Protease™ recognition site (LEVLFQGP). Following fractionation on a Glutathione Sepharose and a Superdex 200 column, the RAD54 pool was incubated with PreScission Protease (GE Healthcare) (10 U/100 mg of RAD54) for 4 h at 4 °C. The sample was diluted to 100 mM KCl and loaded on a 1 ml Resource S column. The fractions containing RAD54 or RAD54 mutants were analyzed for nuclease contamination, pooled, and stored in small aliquots at −80 °C. The protein appeared nearly homogeneous in a Coomassie-stained SDS-polyacrylamide gel (Supplementary Figure 5b).

### Purification of RAD54 and RAD54 mutants from *E. coli*

Human RAD54, RAD54^T31E^, RAD54^S49E^, RAD54^S49A^, RAD54_156–747_, RAD54_1–142_, RAD54_1–142_^T31E^, RAD54 _1–142_^S49E^, RAD54_1–142_^33AAAA36^, RAD54_1–142_^52AA53^, RAD54^33AAAA36^, and RAD54^33AAAA36, S49E^ with a Sumo-His affinity tag, were cloned in the pETHSUL vector^47^ and expressed in *E. coli* Rosetta 2 (DE3) cells. The cDNA sequence used for cloning was amplified using DNA primers (Supplementary Table 2) and pFastBac-HTc vector containing RAD54 cDNA as template. The cloning was performed using ligation-independent cloning protocol^47^. All RAD54 mutants were generated using QuickChange site directed mutagenesis kit (Agilent Technologies) and DNA primers (Supplementary Table 2). Rosetta 2 (DE3) cells were transformed with the recombinant plasmids and cultured at 37 °C to an O.D._600_ of 0.4. The cultures were transferred to 16 °C and allowed to grow until an O.D._600_ of 0.6–0.8, followed by induction of protein expression with 0.1 mM isopropyl-1-thio-β-d-galactopyranoside for 20 h. Cells were harvested by centrifugation (7000×*g*) and stored at −80 °C. All purification steps were carried out at 4 °C. Cells (10 g) were thawed and resuspended in ten volumes of lysis buffer (20 mM KH_2_PO_4_ pH 7.5, 200 mM KCl, 10% sucrose, 10 mM 2-mercaptoethanol) supplemented with EDTA-free protease inhibitor cocktail (Roche Applied Science). The cells were lysed by passing their suspension twice through an Emulsiflex C-5 (Avestin) at 15,000–20,000 psi. The crude extract was clarified by centrifugation (135,000×*g* for 60 min) and passed through 0.45 µm filter. The filtrate was loaded onto a 5 ml HiTrap Ni^2+^ column (GE Healthcare). The column was washed extensively with buffer A (20 mM KH_2_PO_4_ pH 7.5, 500 mM KCl, 10% glycerol, 10 mM 2-mercaptoethanol) supplemented with 50 mM Imidazole. RAD54 protein was eluted using buffer A supplemented with 500 mM imidazole and the eluate was supplemented with 2 mM EDTA. Sumo-His tags were cleaved off by Sumo hydrolase (dtUD1) (200 µg) for 4 h at 4 °C. The cleaved RAD54 was fractionated in a Superdex 200 column (58 ml) equilibrated with buffer A. The fractions containing RAD54 were pooled, diluted five times using buffer B (20 mM KH_2_PO_4_ pH 7.5, 10% glycerol, 10 mM 2-mercaptoethanol) and loaded on to 1 ml Resource S column (GE Healthcare), equilibrated with buffer B supplemented with 100 mM KCl. The proteins were eluted with a 20 ml gradient of KCl (100–450 mM) in buffer B. The fractions containing RAD54 or RAD54 mutants were analyzed for nuclease contamination, pooled, and stored in small aliquots at −80 °C.

The RAD54_1–142_, was purified as above except that a 58-ml Sephacryl S-200 HR column (GE Healthcare) was used in place of Superdex 200. For the RAD54_156–747_ the Resource S step was omitted  due to large protein loses. All proteins, except RAD54_156–747_, appeared nearly homogeneous in a Coomassie-stained SDS-polyacrylamide gel (Supplementary Figure 5b). RAD54_156–747_ contains some contaminating protein bands, but it was free from nuclease contamination.

### Spectrophotometric ATPase assay

The hydrolysis of ATP by Rad54 protein was monitored spectrophotometrically^48^. The oxidation of NADH, coupled to ADP phosphorylation, resulted in a decrease in absorbance at 340 nm, which was continuously monitored by a Hewlett-Packard 8453 diode array spectrophotometer using UV–visible ChemStation software. The rate of ATP hydrolysis was calculated from the rate of change in absorbance using the following formula: rate of *A*_340_ decrease (s^−1^) × 9880 = rate of ATP hydrolysis (µM min^−1^). The reactions were carried out in standard buffer containing 25 mM Tris-acetate, pH 7.5,  3 mM MgCl_2_, 1 mM DTT, 2 mM ATP, 3 mM phosphoenolpyruvate, pyruvate kinase (20 U ml^−1^), lactate dehydrogenase (20 U ml^−1^), and NADH (200 μg ml^−1^) unless otherwise indicated, and the indicated concentrations of RAD54 and DNA. Reactions were carried out at 30 °C unless otherwise indicated.

### BM assay

PX junctions were prepared by annealing ^32^P-labeled forked DNA intermediates (#71/169*, 32 nM) with 3′-tailed DNAs (#170/171, 48 nM) (50% molar excess of cold tailed DNA)^46^. The RAD54 or the RAD54 mutant protein (60 nM, unless indicated otherwise) was incubated with ^32^P-labeled synthetic PX junction (32 nM, molecules unless indicated otherwise), in a 100-µl BM buffer containing 25 mM Tris-acetate, pH 7.5, 3 mM magnesium acetate, 2 mM ATP, 1 mM dithiothreitol, 100 µg ml^−1^ BSA, the ATP-regenerating system (30 U ml^−1^ creatine phosphokinase and 10 mM creatine phosphate). The reactions were carried out at 30 °C. Aliquots (10 µl) were withdrawn at indicated time points, and DNA substrates were deproteinized by treatment with stop solution (1.36% SDS, 1.4 mg ml^−1^ proteinase K, 6% glycerol, 0.015% bromophenol blue) for 15 min at 37 °C. Samples were analyzed by electrophoresis in 8% polyacrylamide gels (29:1) in 1× TBE buffer (90 mM Tris borate, pH 8.3, and 1 mM EDTA) at room temperature. The gels were dried on DE81 chromatography paper (Whatman) and quantified using a Storm 840 PhosphorImager (GE Healthcare).

### Triple-helix displacement assay

SspI-linearized pMJ5 (100 nM, molecules) and ^32^P-labeled triplex-forming oligonucleotide (TFO) (oligo TFO, 22 mer) (100 nM, molecules) were mixed in buffer containing 25 mM MES (pH 5.5), and 10 mM MgCl_2_ and incubated for 15 min at 57 °C, followed by cooling to room temperature and overnight incubation. The RAD54 (5 nM) was incubated for 5 min at 20 °C in 100-µl reaction buffer containing 35 mM Tris-HCl, pH 7.2, 3 mM MgCl_2_, 100 µg ml^−1^ BSA, 1 mM DTT, 2 mM ATP, and the ATP-regenerating system (30 U ml^−1^ creatine phosphokinase and 15 mM creatine phosphate). The reaction was initiated by addition of the triple-helix substrate (pMJ5 + TFO) (0.5 nM, molecules). Overall, 10 µl aliquots were withdrawn at indicated time points and the reaction was quenched by adding 5 µl of stop solution containing 1.36% SDS, 1.4 mg ml^−1^ proteinase K, 6% glycerol, 0.015% bromophenol blue followed by 15 min incubation at 37 °C. The DNA products were analyzed by electrophoresis in 1.2% agarose gels in buffer containing 40 mM Tris-acetate, pH 5.5, 5 mM sodium acetate, and 1 mM MgCl_2_ at 3.5 V cm^−1^ for 2 h at 4 °C. The gels were dried on DE81 chromatography paper (Whatman) and quantified using a Storm 840 PhosphorImager (GE Healthcare).

### Gel retardation assay

The non-mobile ^32^P-labeled PX junction (oligos 174/175/176/181) (30 nM, molecules) was incubated in binding buffer containing 25 mM Tris-acetate (pH 7.5), 5 mM magnesium acetate, 100 µg ml^−1^ BSA, 2 mM DTT, 2 mM ATP, and 10% glycerol for 5 min at 4 °C. Then RAD54 or RAD54 mutants (at indicated concentrations) were added to the reaction mixture followed by additional incubation for 45 min. RAD54–DNA complexes were analyzed by electrophoresis in 6% non-denaturing polyacrylamide gels (29:1) in 0.25× TBE buffer (22.5 mM Tris borate pH 8.3 and 0.5 mM EDTA). The gels were dried on DE81 chromatography paper (Whatman) and the results were quantified using a Storm 840 PhosphorImager (GE Healthcare).

In competition-binding assay, the non-mobile ^32^P-labled PX junction (oligos 174/175/176/181) (30 nM, molecules) was incubated with RAD54_1–142_ (300 nM) in the presence of varying concentrations of non-radioactive DNA competitors, ssDNA (oligo 2), dsDNA (oligos 1/2), PX junction (oligos 174/175/176/181), X-junction (oligos 174/175/176/177), in the binding buffer for 45 min at 4 °C. The protein–DNA complexes were analyzed by electrophoresis in non-denaturing polyacrylamide gels.

### In vitro CDK2 phosphorylation assay

RAD54, RAD54_1–142_, or RAD54^S49A^ (500 ng) was incubated in a 10 µl reaction mixture containing 5 mM HEPES-KOH, pH 7.5, 10 mM MgCl_2_, 1 mM DTT, 30 µM ATP and 83.5 nM [γ-^32^P] ATP (6000 mCi mmole^−1^) with 500 ng of purified CDK2/CyclinE complexes for 30 min at 30 °C. Following this step, the phosphorylated proteins were either used in RAD54 activity assays, or the reactions were stopped by addition of EDTA to 25 mM and equal volume of 2× Laemmli buffer (100 mM Tris-HCl, pH 6.8, 20% glycerol, 2% β-mercaptoethanol, 4% SDS, and 0.02% bromophenol blue) and incubation for 5 min at 75 °C and proteins were analyzed by SDS-PAGE. The gels were stained by Blue protein stain (Denville). The gels were then dried on DE81 chromatography paper and radioactive protein bands were quantified using a Storm 840 PhosphorImager (GE Healthcare). To estimate the extent of RAD54 phosphorylation, we used radioactive standards that were prepared by diluting [γ-^32^P] ATP stock 1000, 5000 and 10,000 times, and applying 1 µl of each dilution onto DE81 chromatography paper that was exposed together with the gel containing phosphorylated RAD54.

### D-loop formation

The nucleoprotein filaments were formed by incubating RAD51 protein with ^32^P-labeled ssDNA (#90, 90-mer) in buffer containing 35 mM Tris-HCl, pH 7.5, 2 mM ATP, 100 µg ml^−1^ BSA, 1 mM DTT, 20 mM KCl (from the protein stock), 1.5 mM MgCl_2_, 0.5 mM CaCl_2_, and the ATP-regenerating system (30 U ml^−1^ creatine phosphokinase and 20 mM creatine phosphate for 30 min at 37 °C. The reaction was transferred to 30 °C followed by addition of RAD54 and supercoiled pUC19 dsDNA (50 µM nucleotide or 9.3 nM molecules) to initiate D-loop formation. Aliquots (10 µl) were withdrawn at indicated time points, and D-loops were deproteinized by treatment with 5 µl of stop solution (1.36% SDS, 1.4 mg ml^−1^ proteinase K, 6% glycerol, 0.015% bromophenol blue) for 15 min at 37 °C; and analyzed by electrophoresis in 1% agarose-TAE (40 mM Tris-acetate, pH 8.0, and 1 mM EDTA) gels. The gels were dried on DE81 chromatography paper and quantified using a Storm 840 PhosphorImager (GE Healthcare).

### ATPase assay using thin layer chromatography (TLC)

The phosphorylated or non-phosphorylated RAD54 protein (60 nM) was incubated with supercoiled pUC19 (2 µM, nt) DNA substrate and 8 mM ATP, 20 nCi of [γ-^32^P]ATP in 10 µl of reaction buffer containing 25 mM Tris-acetate, pH 7.5, 2 mM DTT, 100 μg ml^−1^ BSA, 11 mM magnesium acetate at 30 °C. The level of ATP hydrolysis was determined using TLC on PEI-cellulose plates in running buffer containing 1 M formic acid and 0.3 M LiCl. The products of ATP hydrolysis were quantified using a Storm 840 PhosphorImager (GE Healthcare).

### RAD54 protein cross-linking

RAD54 or RAD54 mutants (1.2 µM) were incubated in 10 µl reaction mixtures containing 20 mM HEPES-KOH, pH 7.5, 5 mM MgCl_2_, 5 mM EDTA, and 40 mM KCl with PX junction (oligos 174/175/176/181) (0.4 µM, molecules) for 5 min at 25 °C. Followed by, addition of bis-maleimidohexane (BMH) (Pierce) to a final concentration of 25 µM. After a 10-min incubation at 25 °C, the reactions were quenched by the addition of β-mercaptoethanol to a final concentration of 1.1 M. The samples were analyzed by electrophoresis in 7.5% or 15% SDS-PAGE gels for RAD54 or RAD54_1–142_ proteins, respectively. The proteins were visualized by silver staining (Invitrogen).

### Data availability

The data that support the findings of this study are available from the corresponding author upon reasonable request.

## Electronic supplementary material

Supplementary Information
