# Supplementary material for: RAD54 N-terminal domain is a DNA sensor that couples ATP hydrolysis with branch migration of Holliday junctions
Source: Nat Commun. 2018 Jan 2;9:34. doi: 10.1038/s41467-017-02497-x (PMC5750232; doi:10.1038/s41467-017-02497-x)
Supplement: Supplementary file 1 — Supplementary Information [file 41467_2017_2497_MOESM1_ESM.pdf]

SUPPLEMENTARY METHODS

Mass spectrometry analysis of *in vitro* phosphorylated GST-RAD54

Kinase assay was carried out using 1 μg of purified recombinant GST-RAD54 and 100 ng of purified recombinant GST-cyclin E/CDK2. The reaction was incubated for 1 h at 30 °C and the protein mixture was denatured by adding acetonitrile to 15% and digested with 0.2 μg trypsin (Promega) for 3 h at 37 °C. Sample was then desalted using a C18 Ziptip (EMD Millipore), and one-quarter of the sample was loaded onto an in-house-packed C18 microcapillary column (75 μm inner diameter x 15 cm bed length) and resolved by a 60 min gradient of 5-28% acetonitrile containing 0.1% formic acid (v/v) at the flow rate of 300 nl/min by nanoflow liquid chromatography (nanoLC) using an Agilent 1100 nano pump with electronically controlled split flow. Tandem mass spectrometry (MS/MS) was carried out using an LTQ-Orbitrap mass spectrometer (ThermoFisher Scientific) using MS resolution of 30, 000 and top MS/MS in the ion-trap. Tandem mass spectra acquired were searched against a human Uniprot database (Version 2012_09) with target-decoy using the Comet algorism [1]. Peptide search parameters included 50ppm parental peptide mass tolerance, one tryptic end, and differential mass modification to methionine (+15.999) due to oxidation and serine and threonine (+79.9663) due to phosphorylation. Search results were filtered using Trans Proteomic Pipeline [2] with peptide false discovery rate (FDR) of 0.9%.

Supplementary References:

1. Eng, J.K., Jahan, T.A. & Hoopmann, M.R. Comet: an open-source MS/MS sequence database search tool. *Proteomics* **13**, 22-4 (2013).
2. Deutsch, E.W. et al. Trans-Proteomic Pipeline, a standardized data processing pipeline for large-scale reproducible proteomics informatics. *Proteomics Clin Appl* **9**, 745-54 (2015).

SUPPLEMENTARY FIGURES

Supplementary Figure 1

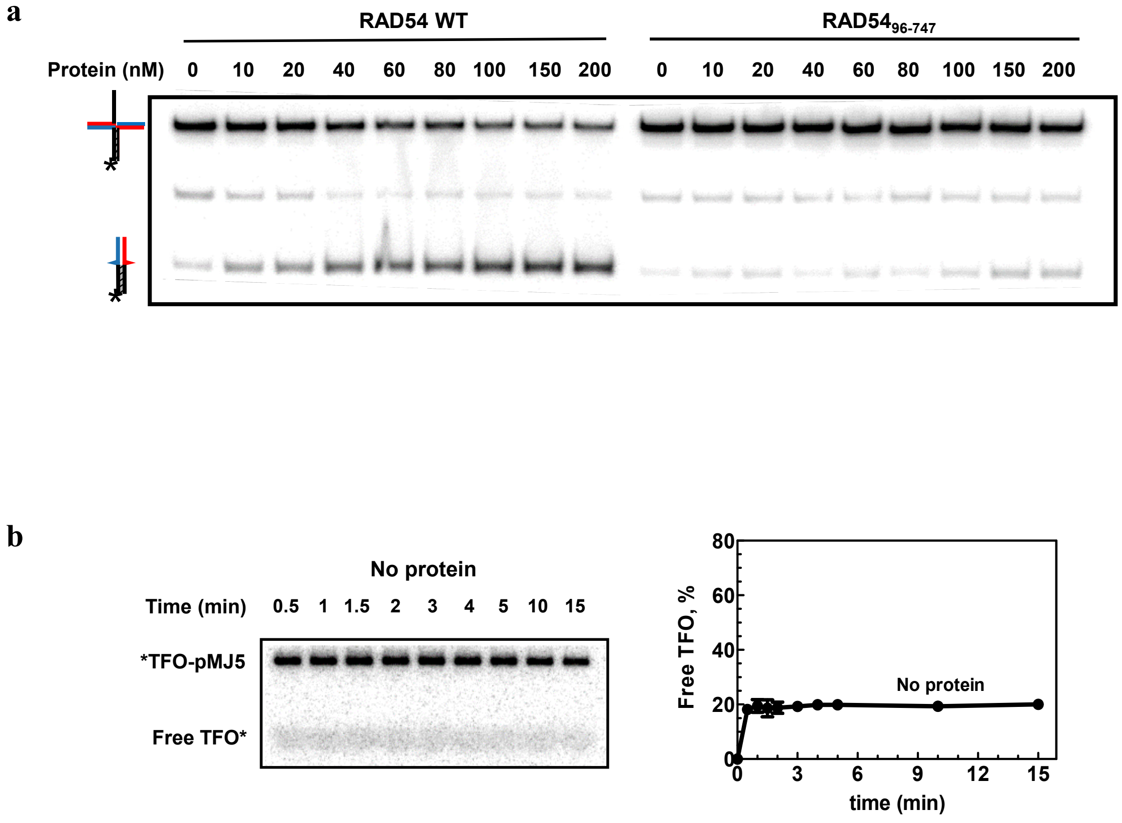

Supplementary Figure 1. $RAD54_{96\text{-}747}$ is deficient in BM activity**.** a) The DNA products of BM reactions promoted by RAD54 or $RAD54_{96\text{-}747}$ on PX-junction (no. 71/169/170/171) (10 nM, molecules) were analyzed by electrophoresis in 8% polyacrylamide gels. b) The kinetics of spontaneous triple-helix (0.5 nM, molecules) displacement was analyzed by electrophoresis in 1.2 % agarose gels. These values have been subtracted from the values reported in Fig. 2c. Each experiment was repeated three times. Error bars represent the s.e.m.

Supplementary Figure 2

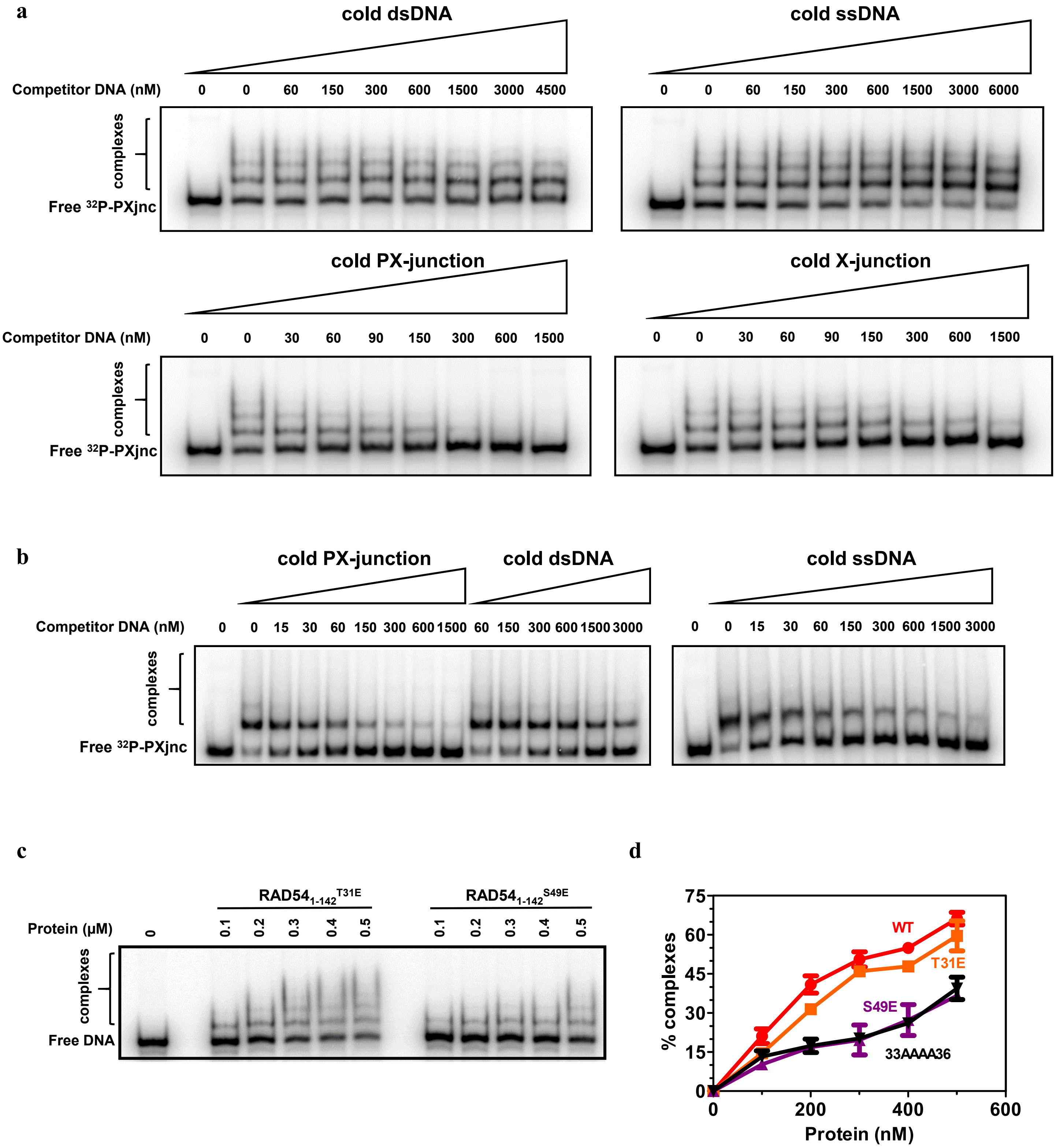

Supplementary Figure 2. DNA binding properties of $RAD54_{1\text{-}142}$. a) $RAD54_{1\text{-}142}$ (300 nM) was incubated with ^{32}P-labeled non-mobile PX junction (no. 174/175/176/181; 30 nM) and indicated concentrations of DNA competitors. The complexes were analyzed using polyacrylamide gels. The gels were quantified and the data presented graphically in Figure 3a. b) $RAD54_{156\text{-}747}$ (100 nM) was incubated with ^{32}P-labeled non-mobile PX junction (no. 174/175/176/181; 30 nM) and indicated concentrations of DNA competitors. The complexes were analyzed using polyacrylamide gels. The gels were quantified and the data presented graphically in Figure 3a. c) The effect of phosphomimetic mutants of $RAD54_{1\text{-}142}$ on binding to PX-junction (30 nM) was analyzed by electrophoresis in 6% polyacrylamide gel. d) Data from C plotted as graph. Each experiment was repeated three times. Error bars represent the s.e.m.

Supplementary Figure 3

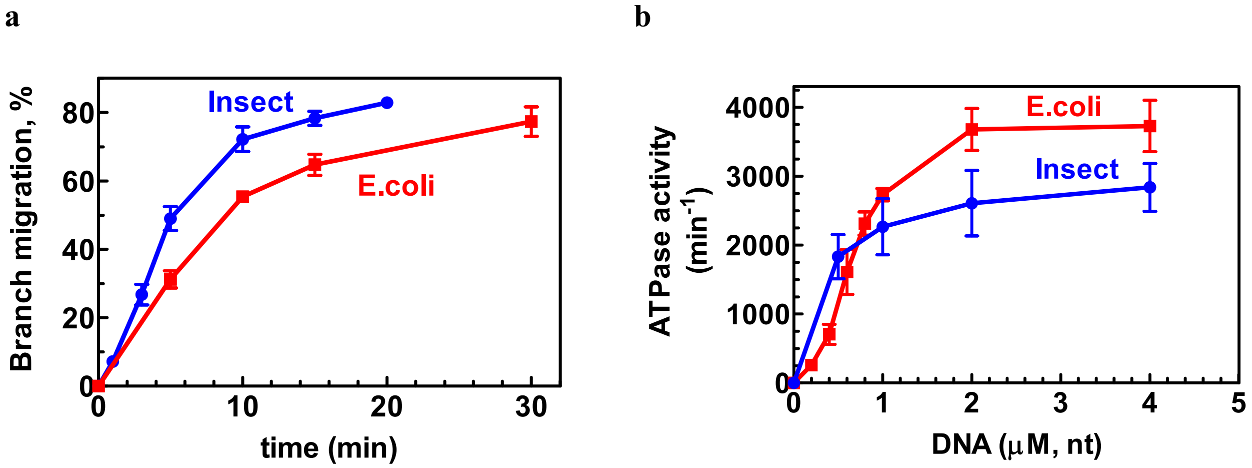

Supplementary Figure 3. Comparing activities of RAD54 purified from insect cells and bacterial cells. a) The kinetics of BM of PX-junction (10 nM) promoted by RAD54 WT (30 nM) purified from insect or *E.coli* cells. The BM products were analyzed by electrophoresis in 8% polyacrylamide gels. b) ATP hydrolysis by RAD54 WT (20 nM) purified form insect cells or from *E.coli* using supercoiled pUC19 as DNA substrate. Each experiment was repeated three times. Error bars represent the s.e.m.

Supplementary Figure 4

a

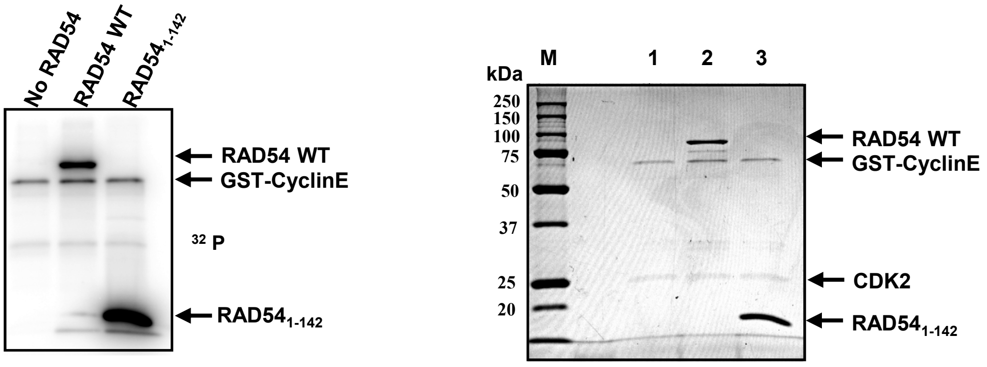

b

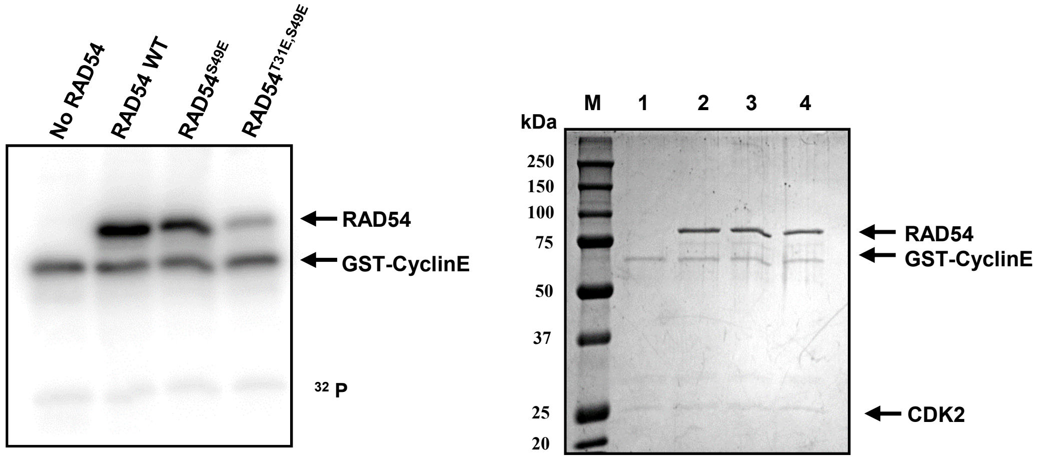

Supplementary Figure 4. CDK2 phosphorylates the N-terminal region of RAD54 *in vitro*. a) *Left panel*, Autoradiograph showing *in vitro* phosphorylation of RAD54 (500 ng) and $RAD54_{1-142}$ (500 ng) in the presence of [γ-^{32}P] ATP with purified CDK2/cyclin complex. *Right panel*, The same gel stained by Coomassie G250. b) *Left panel*, Autoradiograph showing *in vitro* phosphorylation of RAD54 WT (500 ng), $RAD54^{S49E}$ (500 ng), and $RAD54^{T31E,\ S49E}$ (500 ng) with CDK2/cyclin complex using [γ-^{32}P] ATP. *Right panel*, The same gel stained by Coomassie G250.

Supplementary Figure 5

a

T31 peptides

R.SCDDEDWQPGLVTPR.K (0)
R.SCDDEDWQPGLVT*PR.K (11)
C.DDEDWQPGLVT*PR.K (1)
R.S*CDDEDWQPGLVT*PR.K (2)
Ratio of phosphorylated vs. unphosphorylated T31: 14:0

S49 Peptides

K.SSSETQIQECFLSPFR.K (2)
R.KSS*SETQIQECFLSPFR.K (1)
K.SSSETQIQECFLS*PFR.K (5)
K.SSS*ETQIQECFLS*PFR.K (1)
R.KSSSETQIQECFLS*PFR.K (2)
R.KSS*SETQIQECFLS*PFR.K (2)
R.KSSS*ETQIQECFLS*PFR.K (1)
K.RKS*SSETQIQECFLS*PFR.K (1)
K.RKSSS*ETQIQECFLS*PFR.K (1)
K.SSSETQIQECFLS*PFRKPLSQLTNQPPCLDSSQHEAFIR.S (1)
R.KS*SSETQIQECFLS*PFRKPLSQLTNQPPCLDSSQHEAFIR.S (2)
Ratio of phosphorylated vs. unphosphorylated S49: 16:3

b

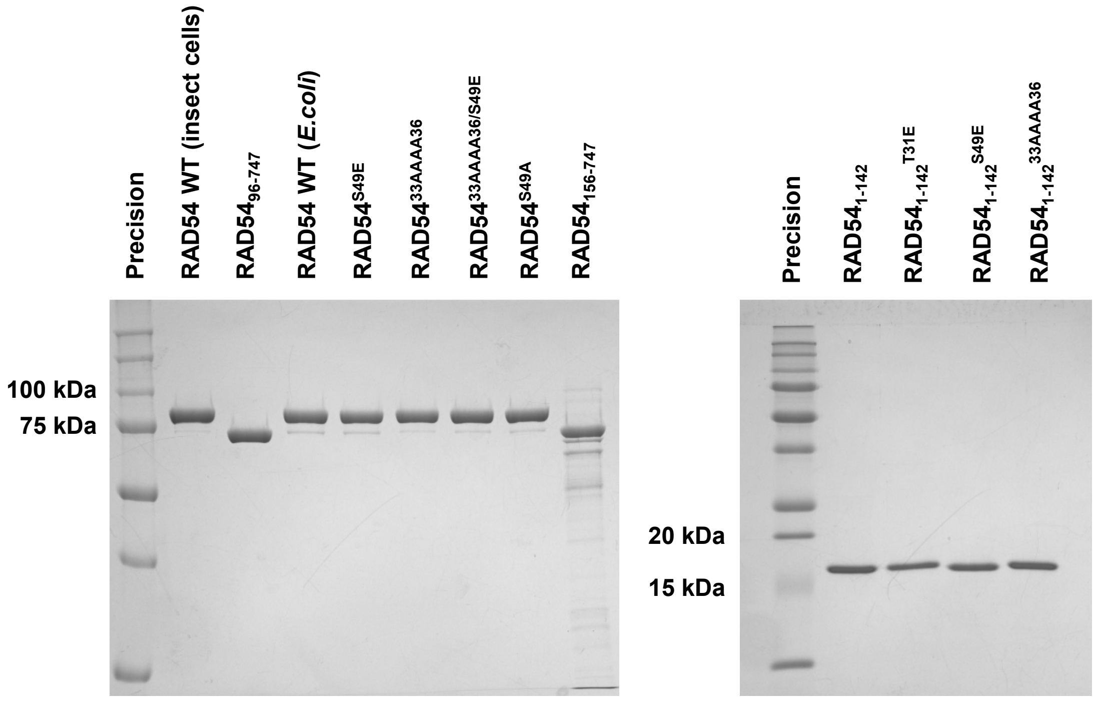

Supplementary Figure 5. a) Phosphorylation of GST-RAD54 *in vitro* by GST-CDK2/Cyclin E. The mass spectrometric analysis of *in vitro* phosphorylation of RAD54 by CDK2. GST-RAD54 (1 μg) was incubated with GST-cyclin E/CDK2 (100 ng) for 1 h at 30 °C, followed by denaturation and digestion by trypsin (0.2 μg) for 3 h at 37 °C. The peptides were then analyzed by NanoLC-electrospray ionization (ESI) - MS/MS using an LTQ-Orbitrap mass spectrometer. Asterisks mark the phosphorylated residues. Numbers in parentheses denote the peptide spectral count. b) The purified recombinant proteins (1μg) used in this study analyzed on either 10% (left panel) or 15% (right panel) SDS-PAGE.

Supplementary Table 1. Sequences of the oligonucleotides used in this study *

Number	Length in nucleotides	Sequence, 5'→3'
#64	48	GTCGACGACGTCTGAGTACTCATCTAGTGTGACATCATCGCATCGAGA
#65	48	TCTCGATGCGATGATGTCACACTAGATGAGTACTCAGACGTCGTCGAC
#71	94	CTTTAGCTGCATATTTACAACATGTTGACCTACAGCACCAGATT**C**AGC AATTAAGCTCTAAGCCATCCGCAAAAATGACCTCTTATCAAAAGGA
#169	93	TCCTTTTGATAAGAGGTCATTTTTGCGGATGGCTTAGAGCTTAATTGC T**G**AATCTGGTGCTGTTTTTTTTTTTTTTTTTTTTTTTTTTTTTTT
#170	94	TCCTTTTGATAAGAGGTCATTTTTGCGGATGGCTTAGAGCTTAATTGC T**A**AATCTGGTGCTGTAGGTCAACATGTTGTAAATATGCAGCTAAAG
#171	63	ACAGCACCAGATT**T**AGCAATTAAGCTCTAAGCCATCCGCAAAAATGAC CTCTTATCAAAAGGA
#174	61	GACGCTGCCGAATTCTACCAGTGCCTTGCTAGGACATCTTTGCCCACC TGCAGGTTCACCC
#175	62	TGGGTGAACCTGCAGGTGGGCAAAGATGTCCTAGCAATGTAATCGTCA AGCTTTATGCCGTT
#176	63	CAACGGCATAAAGCTTGACGATTACATTGCTAGGACATGCTGTCTAGA GGATCCGACTATCGA
#177	62	ATCGATAGTCGGATCCTCTAGACAGCATGTCCTAGCAAGGCACTGGTA GAATTCGGCAGCGT
#180	25	CTTTGCCCACCTGCAGGTTCACCCA
#181	25	TCGATAGTCGGATCCTCTAGACAGC
#244	60	ACATTGCTAGGACATGCTGTCTAGAGGATCCGACTATCGATAAAACCC TGCAAGTTCGTA
#249	30	TGCAGGTGGGCAAAGATGTCCTAGCAATGT
#250	15	CTTTGCCCACCTGCA
TFO	22	TTCTTTTCTTTCTTCTTTCTTT

***Bold red letters** indicate the nucleotides that form mismatched pairs in the branch migration products.

Supplementary Table 2. Primers used in this study *

	Forward/ Reverse	Sequence, 5'→3'
RAD54 WT	Forward	AGATTGGTGGCGGCATGAGGAGGAGCTTGGCTCCC
	Reverse	GAGGAGAGTTTAGACATTAGCGGAGGCCCCGCTGTTCCTCATG
N-terminal RAD54	Forward	AGATTGGTGGCGGCATGAGGAGGAGCTTGGCTCCC
	Reverse	GAGGAGAGTTTAGACATTAATGGACAGGGAGTTTCTCCTTG
RAD54 S49E	Forward	GGAGTGTTTCCTG**GAA**CCTTTTCGGAAACCTTTGAG
	Reverse	CTCAAAGGTTTCCGAAAAGG**TTC**CAGGAAACACTCC
RAD54KRK3A(residues 12-14)	Forward	CCCAGCCAGCTGGCC**GCGGCAGCA**CCTGAAGGCAGGTCC
	Reverse	GGACCTGCCTTCAGG**TGCTGCCGC**GGCCAGCTGGCTGGG
RAD54RKRK4A (residues (33-36)	Forward	CCTGGCCTAGTGACTCCT**GCGGCAGCGGCA**TCCAGCAGTGAGACCC
	Reverse	GGGTCTCACTGCTGGA**TGCCGCTGCCGC**AGGAGTCACTAGGCCAGG
RAD54RK2A (residues 52-53)	Forward	GAGTGTTTCCTGTCTCCTTTT**GCGGCA**CCTTTGAGTCAGCTAACC
	Reverse	GGTTAGCTGACTCAAAGG**TGCCGC**AAAAGGAGACAGGAAACACTC

***Bold red letters** indicate the nucleotides that were mutated.
